# Phosphated cyclodextrins as water-soluble chiral NMR solvating agents for cationic compounds

**DOI:** 10.3762/bjoc.13.6

**Published:** 2017-01-06

**Authors:** Cira Mollings Puentes, Thomas J Wenzel

**Affiliations:** 1 Department of Chemistry, Bates College, Lewiston, Maine 04240 USA

**Keywords:** chiral, chiral differentiation, cyclodextrin, enantiomer, enantiomeric purity, NMR

## Abstract

The utility of phosphated α-, β- and γ-cyclodextrins as water-soluble chiral NMR solvating agents for cationic substrates is described. Two sets of phosphated cyclodextrins, one with degrees of substitution in the 2–6 range, the other with degrees of substitution in the 6–10 range, are examined. Results with 33 water-soluble cationic substrates are reported. We also explored the possibility that the addition of paramagnetic lanthanide ions such as praseodymium(III) and ytterbium(III) further enhances the enantiomeric differentiation in the NMR spectra. The chiral differentiation with the phosphated cyclodextrins is compared to prior results obtained with anionic carboxymethylated cyclodextrins. There are a number of examples where a larger differentiation is observed with the phosphated cyclodextrins.

## Introduction

Chiral NMR solvating agents are commonly used for determining enantiomeric purity. In some cases, these compounds cause reproducible perturbations in chemical shifts that can be used in the assignment of the absolute stereochemistry [1–7]. Since chiral solvating agents associate with the compound being studied through non-covalent interactions, they are easy to use and involve merely mixing the reagent with the compound in an NMR tube.

**Figure 1:**
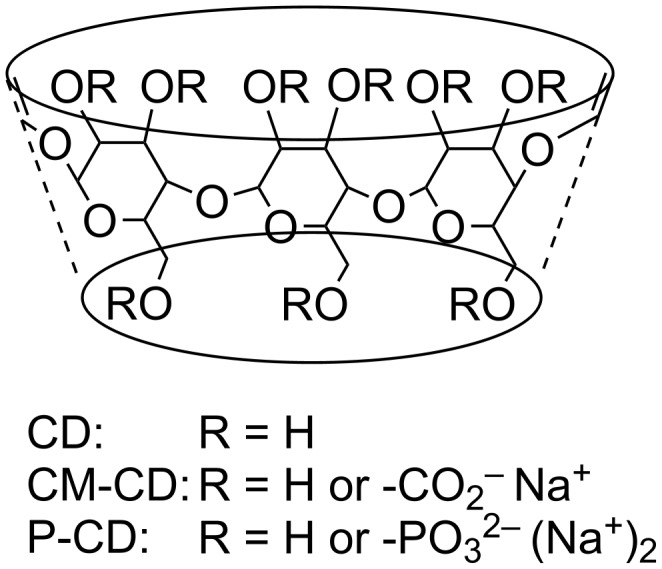
Structure of cyclodextrins (CD) and their carboxymethylated (CM-CD) and phosphated (P-CD) derivatives.

Cyclodextrins (CDs), which are cyclic oligosaccharides containing D-glucose units, represent an important and versatile class of chiral NMR solvating agents (Figure 1). The most common representatives are α-, β- and γ-CD, which contain six, seven and eight glucose rings, respectively. Many substrates form inclusion complexes with CDs and the differing sizes of α-, β- and γ-CD allow the study of substrates of different sizes. Furthermore, the secondary hydroxy groups at the 2 and 3-positions and the primary hydroxy groups at the 6-position of CDs can be derivatized with a variety of functional groups. Such derivatization can be used to alter the solubility, binding properties of substrates, and ultimately enantioselectivity properties of the CDs. The cavity of CDs has the secondary hydroxy groups at one opening and the primary ones at the other and the opening to the cavity at the secondary side is larger than that at the primary side.

Native, underivatized CDs are effective chiral NMR solvating agents in water [8–9]. Water-soluble substrates with hydrophobic moieties such as aryl rings typically form inclusion complexes by insertion of the aryl ring into the CD cavity. Neutral CDs with permethylated [10–12], benzylated [13], benzoylated [13–14], carbamoylated [15–18], and acetylated [19–20] hydroxy groups have been studied in NMR applications. The permethylated CDs can be used in both aqueous and organic solvents whereas the other neutral derivatives are typically useful in organic solvents such as chloroform-*d*. In addition to the aforementioned modifications, the hydroxy groups of CDs can also be derivatized with ionic substituents. CDs with anionic carboxymethyl [21–32] (CM-CD, Figure 1), sulfate [29–30,33–35], sulfobutylether [22–24,36–37] and thiocarboxymethyl [38] groups also have been studied as chiral NMR solvating agents. The general findings of these studies are that anionic CDs are more effective chiral NMR solvating agents for cationic substrates than neutral native CDs. Similarly cationic CDs containing amine [39–40], xylylenediamine [41], and trialkylammonium groups [42–44] are found to be more effective for anionic substrates than the neutral native CDs.

An important consideration with derivatized CDs is the degree of substitution (DS) of the hydroxy groups. When preparing ionic CD derivatives, it is often difficult if not impossible to derivatize all of the hydroxy groups. Whether the functionalization takes place preferentially at the primary or secondary hydroxy groups can have a significant impact on the enantioselectivity of the resulting derivative. Carboxymethyl- and trimethylammonio-substituted CDs are commercially available but often have a low DS of about 2. Previous reports have found that randomly substituted ionic CDs with higher degrees of carboxymethylation and trimethylammonium groups (DS = 11 for β-CD) [21,43] are considerably more effective than derivatives with lower DS. Unfortunately, CM-CDs with high DS are not commercially available and investigators wishing to use these compounds in chiral NMR applications would have to synthesize and purify them.

Phosphated CD derivatives (P-CDs, Figure 1) have been utilized as effective enantioselectors in capillary electrophoresis [45–48]. Various phosphated α-, β- and γ-CD are commercially available with different degrees of substitutions from low (2–6) to high DS (6–10). This report will describe the utilization of P-CDs as water-soluble chiral NMR solvating agents. Thirty-three cationic substrates with a wide range of structural features are examined and enantiomeric differentiation obtained with P-CDs is compared to prior results acquired with CM-CDs.

## Results and Discussion

**Figure 2:**
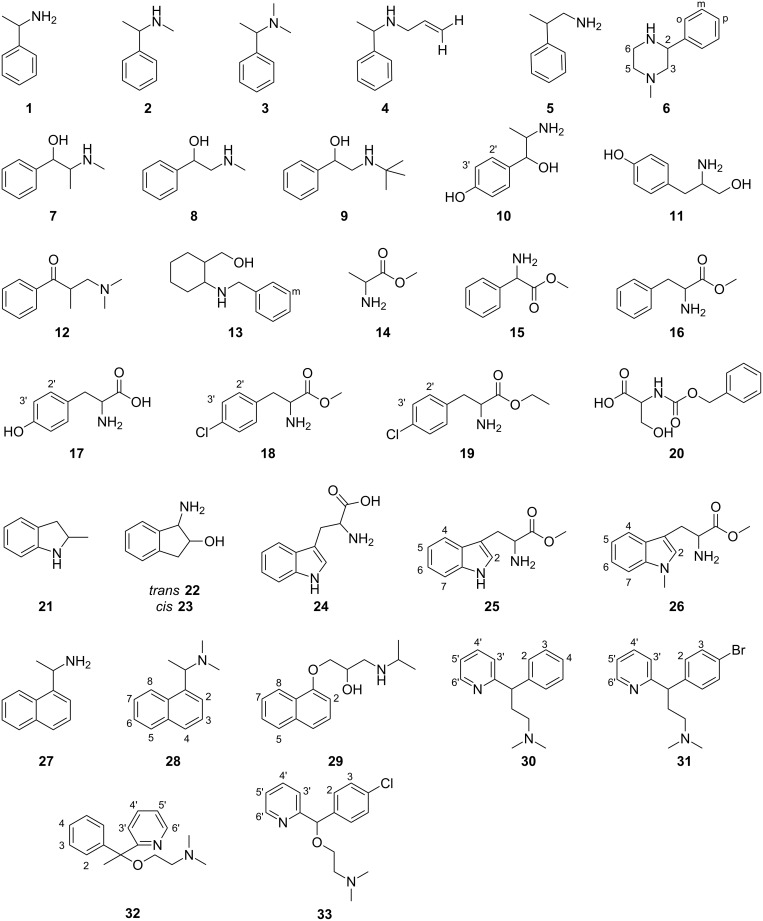
Structures of substrates included in the study. All compounds were examined in their protonated forms with the P-CDs.

Thirty-three substrates including α-methylbenzylamine (**1**), *N*,α-dimethylbenzylamine (**2**), *N*,*N*-dimethyl-1-phenethylamine (**3**), *N*-allyl-α-methylbenzylamine (**4**), β-methylphenethylamine (**5**), 1-methyl-3-phenylpiperazine (**6**), ephedrine (**7**), α-(methylaminoethyl)benzyl alcohol (**8**), 2-*tert*-butylamino-1-phenylethanol (**9**), α-(1-aminoethyl)-4-hydroxybenzyl alcohol (**10**), tyrosinol (**11**), 3-dimethylamino-2-methylpropiophenone (**12**), *cis*-(2-benzylamino)cyclohexanemethanol (**13**), alanine methyl ester (**14**), 2-phenylglycine methyl ester (**15**), phenylalanine methyl ester (**16**), tyrosine (**17**), 4-chlorophenylalanine methyl ester (**18**), 4-chlorophenylalanine ethyl ester (**19**), carbobenzyloxy serine (**20**), 2-methylindoline (**21**), *trans*-1-amino-2-indanol (**22**), *cis*-1-amino-2-indanol (**23**), tryptophan (**24**), tryptophan methyl ester (**25**), 1-methyltryptophan methyl ester (**26**), 1-(1-naphthyl)ethylamine (**27**), *N*,*N*-dimethyl-1-(1-naphthyl)ethylamine (**28**), propranolol (**29**), pheniramine (**30**), brompheniramine (**31**), doxylamine (**32**), and carbinoxamine (**33**) (Figure 2) in their protonated cationic form were individually tested with six different P-CDs at cyclodextrin concentrations of 5, 10 and 20 mM.

All substrates, except for **14**, have aromatic rings in their structures. Previous studies [21,31–32] and observations made herein with the P-CDs indicate that host–guest complexes through insertion of the aryl ring into the cavity of the CDs occur which is supported by NMR measurements. In the NMR spectra the resonances of the P-CD H3 and H5 protons, which are located inside the CD cavity, are perturbed to lower frequencies in the spectra due to shielding of these protons by the inserted aryl ring. The largest magnitude of enantiomeric differentiation observed in the ^1^H NMR spectra of the substrates with each P-CD at 5, 10 or 20 mM (P-α-CD-L_DS_, DS = 2–6; P-α-CD-H_DS_, DS = 6–10; P-β-CD-L_DS_, DS = 2–6; P-β-CD-H_DS_, DS = 6–10; P-γ-CD-L_DS_, DS = 2–6; P-γ-CD-H_DS_, DS = 6–10) is reported herein.

Equation 1 and Equation 2 show the association of the (*R*)- and (*S*)-enantiomers of a substrate (Sub) with a chiral solvating agent (CSA).

[1]
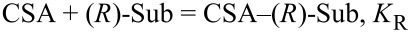


[2]
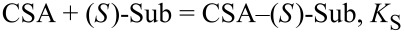


Provided that the exchange of substrates with the CSA is fast on the NMR time scale, there are two mechanisms through which chiral solvating agents (CSAs) can cause enantiomeric differentiation. The first is that the complexes of the two enantiomers of the substrate with the enantiomerically pure CSA are diastereomers and therefore exhibit different chemical shifts. The second relies on a frequently observed difference in the association constants of the two enantiomers with the CSA (K*_R_* and K*_S_*). Under the conditions of fast exchange, one of the enantiomers will preferentially bind with the CSA compared to the other and the time-averaged solvation of the two enantiomers will be different. It is often not possible to determine which mechanism dominates when enantiomeric differentiation is observed in the NMR spectrum, and in many cases both mechanisms likely contribute to some extent.

In cases where enantiomeric differentiation occurs through the formation of diastereomeric CD–substrate complexes, best results in the NMR spectrum would be expected at 20 mM P-CD because of a higher degree of complexation. However, in some spectra there have been an overlap of one of the substrate resonances with other resonances of the substrate or CD in such a way that it was not possible to determine in a regular one-dimensional NMR spectrum whether enantiomeric differentiation was present.

In those cases where enantiomeric differentiation occurs solely through differences in association constants of the two enantiomers of the substrate with the P-CD, the magnitude of the enantiomeric differentiation may decrease at increasing P-CD concentrations from 5 to 20 mM. In this situation, the enantiomer of the substrate with the higher association constant has a higher proportion complexed with the P-CD at 5 mM than the substrate enantiomer with the lower association constant. Therefore, resonances of the substrate with the higher association constant are more perturbed in the NMR spectrum. At higher concentrations of P-CD (10 or 20 mM), also a higher proportion of the enantiomer with the lower association constant binds to the P-CD, thus enhancing perturbations in the NMR spectrum of this enantiomer and thereby diminishing the extent of enantiomeric differentiation. In some cases, the position of the resonances of the two enantiomers in the NMR spectrum may reverse their order as the concentration of the CSA is raised from low to high values. A detailed analysis of this situation has been reported in the literature [49].

**Figure 3:**
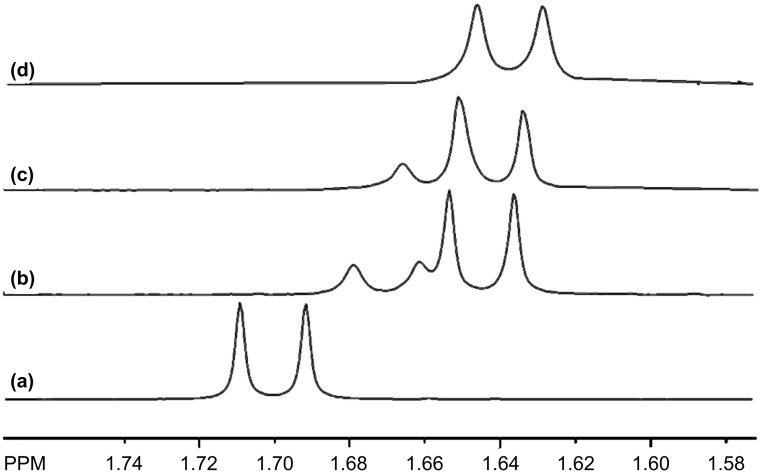
The (a) *C*-methyl resonance of **3** (10 mM, enriched in the (*R*)-enantiomer) in the presence of (b) P-α-CD-H_DS_ (10 mM), (c) P-β-CD-H_DS_ (10 mM) and (d) P-γ-CD-H_DS_ (10 mM).

Substrates **1**–**6** contain amine and aryl moieties. An enantiomeric differentiation is observed in the ^1^H NMR spectra of **1**–**3** in the presence of P-CD, whereas no differentiation is observed in the spectra of **4**–**6** with any of the P-CDs (Table 1). Table 1 and others herein also provide data for enantiomeric differentiation in the spectra of **1**–**6** that was previously reported with a series of carboxymethylated cyclodextrins (CM-CD) [21,31–32]. The differentiation only occurs in the aliphatic resonances of **1**–**6** with P-CDs and CM-CDs. The degree of enantiomeric differentiation in the spectra of **1**–**3** with the different P-CDs show that there is no consistent trend as to which P-CD derivative is more effective at causing enantiomeric differentiation. P-α-CD-H_DS_ is especially effective for substrate **3**, but it is ineffective for the other substrates in this group. P-β-CD-L_DS_ is the only P-CD that is effective for **2**, whereas P-γ-CD-H_DS_ is the only one effective for **1**. Figure 3 shows a comparison of the *C*-methyl resonance of **3** (10 mM) in the presence of P-α-CD-H_DS_, P-β-CD-H_DS_, and P-γ-CD-H_DS_ at a concentration of 10 mM. The most significant degree of enantiomeric differentiation in the spectrum with P-α-CD-H_DS_ is apparent (Figure 3b), as is the smaller differentiation with P-β-CD-H_DS_ (Figure 3c) and the non-existent differentiation with P-γ-CD-H_DS_ (Figure 3d). While CM-CDs causes greater enantiomeric differentiation of more resonances in the NMR spectra of **1**–**6**, there are only a few examples where the P-CDs are more effective. The enantiomeric differentiation of the methine and methyl resonances of **3** with some of the P-CDs is noteworthy.

**Table 1:** Enantiomeric differentiation in ppm in the ^1^H NMR spectra (400 MHz) of **1**–**6** (10 mM) with P-CDs and CM-CD [31–32] in D_2_O. The concentration of the cyclodextrin is 20 mM unless otherwise indicated.

		P-α-CD-L_DS_	P-α-CD-H_DS_	P-β-CD-L_DS_	P-β-CD-H_DS_	P-γ-CD-L_DS_	P-γ-CD-H_DS_	CM-CD

**1**	CH	0	0	0	0	0	0.004	0.010 - β
	CH_3_	0	0	0	0	0	0.007	0
**2**	CH	0	0	0.008	0	0	0	0
	N-CH_3_	0	0	0.006	0	0	0	0.013 - β
	C-CH_3_	0	0	0	0	0	0	0.008 - β
**3**	CH	0.040^a^	0.048^a^	0	0	0	0	0
	C-CH_3_	0.029^b^	0.027^a^	0.016	0.026	0.027	0	0.010 - β
**4**	CH_3_	0	0	0	0	0	0	0.010 - α
**5**	CH_3_	0	0	0	0	0	0	0.007 - β
**6**	CH_3_	0	0	0	0	0	0	0.008 - β

^a^10 mM; ^b^5 mM.

**Figure 4:**
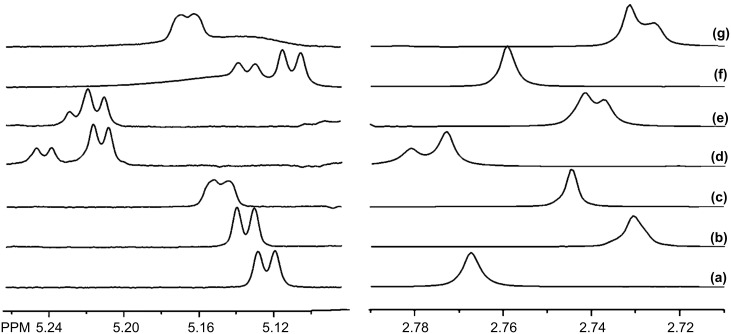
The (a) methine resonance of the carbinol carbon (5.124 ppm) and *N*-methyl resonance (2.768 ppm) of **7** (10 mM, enriched in (1*S*,2*R*)-enantiomer) with 20 mM of (b) P-α-CD-L_DS_, (c) P-α-CD-H_DS_, (d) P-β-CD-L_DS_, (e) P-β-CD-H_DS_, (f) P-γ-CD-L_DS_ and (g) P-γ-CD-H_DS_.

Another group of tested substrates contains aryl, amine and either hydroxy (**7**–**11**, **13**) or carbonyl (**12**) moieties. Within this group, the NMR spectra of **7**, **9** and **10** exhibit enantiomeric differentiation in the presence of one or more of the P-CDs (Table 2). Of particular note is the effectiveness of P-β-CD-L_DS_ for **7** as the CH and CHOH resonances exhibit enantiomeric differentiation on the order of 0.03 ppm. As with compounds **1**–**6**, none of the P-CDs is consistently most effective, either among the different cavity sizes between the α-, β- and γ-P-CDs or among the low and high-DS derivatives. Figure 4 shows a comparison of the methine resonance of the carbinol carbon and the *N*-methyl resonance of **7** with the six different P-CDs. The pronounced enantiomeric differentiation of the methine resonance with P-β-CD-L_DS_ and P-γ-CD-L_DS_ in Figure 4d and f, respectively, is apparent. The smaller enantiomeric differentiation with the P-β-CD-L_DS_ and P-β-CD-H_DS_ and the absence of enantiomeric differentiation of the methine resonance with P-α-CD-L_DS_ and P-α-CD-H_DS_ is also apparent in the spectra shown in Figure 4. For the *N*-methyl resonance, the largest enantiomeric differentiation is also observed with P-β-CD-L_DS_, whereas no enantiomeric differentiation is observed with P-γ-CD-L_DS_. Another interesting observation is that P-β-CD-H_DS_ and P-γ-CD-H_DS_ cause partial enantiomeric differentiation of the *N*-methyl resonance, but the order of the two enantiomers in the spectrum ((1*S,*2*R*) more shielded) is different from that with P-β-CD-L_DS_ ((1*R,*2*S*) more shielded). The use of P-CDs and CM-CDs for **7**–**10** is complementary as several of the resonances show larger enantiomeric differentiation in the NMR spectra with one of the CM-CDs, whereas others are more differentiated with one of the P-CDs.

**Table 2:** Enantiomeric differentiation in ppm in the ^1^H NMR spectra (400 MHz) of **7**–**10** (10 mM) with P-CDs and CM-CD [32] in D_2_O. The concentration of the cyclodextrin is 20 mM unless otherwise indicated.

		P-α-CD-L_DS_	P-α-CD-H_DS_	P-β-CD-L_DS_	P-β-CD-H_DS_	P-γ-CD-L_DS_	P-γ-CD-H_DS_	CM-CD

**7**	CH	0	0	0.034	0.026	0	0	0
	CH-OH	0	0	0.030	0.010	0.024	0	0
	N-CH_3_	0	0	0.008	0.004^a^	0	0.006^b^	0.010 - β
	C-CH_3_	0	0	0.005	0	0	0	0
**8**	N-CH_3_	0	0	0	0	0	0	0.015 - β
**9**	CH-OH	0	0	0	0.025	0	0	
	CH_2_	0	0	0.008	0	0	0	
	CH_2_’	0	0.009	0.008	0	0	0	
**10**	CH-OH	0.006^b^	0	0	0	0	0	0
	CH_3_	0	0	0	0.004	0	0	0.008 - α
	H2’	0	0	0	0	0	0	0.021 - β
	H3’	0	0	0	0	0	0	0.014 - β

^a^5 mM; ^b^10 mM.

Substrates **14**–**20** are a series of amino acids either in their ester (**14**,**16**, **18**, **19**) or acid (**17**, **20**) form. At least one resonance of **14**–**20** exhibits enantiomeric differentiation in the presence of one or more of the P-CDs (Table 3). In most cases, the enantiomeric differentiation is rather minimal with the P-CDs. However, some exceptions include the aryl hydrogen resonances of **19** with P-β-CD-L_DS_ and P-β-CD-H_DS_, one of the methylene resonances of **19** with P-γ-H_DS_, and the methine resonances of **16** with P-γ-CD-L_DS_. The difference in the enantiomeric differentiation of the aryl resonances of **18** and **19** with P-β-CD-L_DS_ and P-β-CD-H_DS_ is noteworthy, as the only difference between the two substrates is that **19** is the ethyl ester and **18** is the methyl ester of 4-chlorophenylalanine. The small change from a methyl to ethyl group obviously has a significant, favorable influence on the enantiomeric differentiation of the aryl resonances while having considerably less effect on the other hydrogen resonances of **18** and **19**. Five of the substrates reported in Table 3 were previously examined with CM-CDs and while one of the CM-CDs is often more effective than the P-CDs, there are examples of resonances of **15**, **16**, **18** and **20** where one of the P-CDs causes the largest enantiomeric differentiation.

**Table 3:** Enantiomeric differentiation in ppm in the ^1^H NMR spectra (400 MHz) of **14**–**20** (10 mM) with P-CDs and CM-CD [31] in D_2_O. The concentration of the cyclodextrin is 20 mM unless otherwise indicated.

		P-α-CD-L_DS_	P-α-CD-H_DS_	P-β-CD-L_DS_	P-β-CD-H_DS_	P-γ-CD-L_DS_	P-γ-CD-H_DS_	CM-CD

**14**	CH	0.007^a^	0	0	0	0	0	
**15**	CH	0	0	0	0	0	0	0.019 - α
	O-CH_3_	0	0	0	0.007^b^	0	0	0
**16**	CH	0	0	0	0	0.044	0	0.016 - α
	CH_2_	0	0	0	0	0	0	0.007 - α
	O-CH_3_	0.002	0.002	0.003	0.005	0	0.003	0.012 - β
**17**	CH	0	0	0	0	0.004^a^	0	0.019 - α
	CH_2_	0.007	0	0	0	0.003^a^	0	0.009 - α
	H2’	0	0	0	0	0	0	0.006 - β
	H3’	0	0	0	0	0	0	0.005 - β
**18**	CH	0	0	0	0.004^a^	0	0	0.004 - β
	CH_2_	0	0	0	0.009^a^	0	0	0.008 - β
	O-CH_3_	0.005^a^	0	0.004^a^	0.004^a^	0.004^a^	0.004^a^	0.012 - β
	H2’	0	0.010	0.009	0.009^a^	0	0	0.006 - β
	H3’	0	0.009	0	0	0	0	0.009 - β
**19**	CH	0.004	0	0	0.003^a^	0	0	
	CH_2_	0	0	0	0.005^a^	0	0.035^a^	
	CH_2_’	0.005	0	0	0	0	0	
	CH_3_	0	0	0	0.002	0	0	
	H2’	0	0	0.021	0.021	0	0	
	H3’	0	0	0.037^a^	0.034	0	0	
**20**	Ar-CH_2_	0.013^a^	0.014	0.006	0	0	0	0

^a^10 mM; ^b^5 mM.

Substrates **21**–**26** contain bicyclic indoline, indane and indole rings. The P-CDs were only effective at causing enantiomeric differentiation in the ^1^H NMR spectra of a few resonances of **21**, **24** and **26** (Table 4). With only two exceptions, the methylene resonance of **24** with P-α-CD-H_DS_ and the *O*-methyl resonance of **26** with P-γ-CD-H_DS_ and P-β-CD-L_DS_, the CM-CDs are more effective at causing enantiomeric differentiation in the ^1^H NMR spectra of the substrates **21**–**26**.

**Table 4:** Enantiomeric differentiation in ppm in the ^1^H NMR spectra (400 MHz) of **21** and **24**–**26** (10 mM) with P-CDs and CM-CD [31] in D_2_O. The concentration of the cyclodextrin is 20 mM unless otherwise indicated.

		P-α-CD-L_DS_	P-α-CD-H_DS_	P-β-CD-L_DS_	P-β-CD-H_DS_	P-γ-CD-L_DS_	P-γ-CD-H_DS_	CM-CD

**21**	CH_2_	0.007	0.002	0	0	0	0	0.009 - β
	CH_3_	0.005	0	0	0	0.009^a^	0	0.015 - β
**24**	CH_2_	0.004^b^	0.007^a^	0	0.004^b^	0	0	0
**25**	CH	0	0	0	0	0	0	0.036 - γ
	CH_2_	0	0	0	0	0	0	0.013 - α
	H2	0	0	0	0	0	0	0.010 - γ
	H4	0	0	0	0	0	0	0.008 - γ
	H5	0	0	0	0	0	0	0.019 - β
	H6	0	0	0	0	0	0	0.019 - β
	H7	0	0	0	0	0	0	0.022 - β
**26**	CH	0	0	0	0	0	0	0.016 - β
	O-CH_3_	0	0	0.010^b^	0	0	0.012^b^	0
	H2	0	0	0	0	0	0	0.011 - β
	H5	0	0	0	0	0	0	0.019 - β
	H6	0	0	0	0	0	0	0.019 - β
	H7	0	0	0	0	0	0	0.020 - β

^a^10 mM; ^b^5 mM.

Substrates **27**–**29** contain naphthyl rings. Naphthyl-containing compounds form inclusion complexes more favorably with the larger β- and γ-cyclodextrins so the general ineffectiveness of P-α-CD-L_DS_ and P-α-CD-H_DS_ for **27**–**29** is not surprising (Table 5). Both the P-β-CDs and P-γ-CDs are effective at causing enantiomeric differentiation in resonances of **27**–**29**. An interesting observation is the ineffectiveness of P-γ-CD-H_DS_ at causing enantiomeric differentiation of in the spectrum of **28**, whereas the P-γ-CD-L_DS_ causes enantiomeric differentiation of four resonances. Similarly, enantiomeric differentiation of the *C*-methyl resonance of **29** with P-β-CD-L_DS_ is much larger (0.027 ppm) than that with P-β-CD-H_DS_ (0.009 ppm). Substrate **29** is also examined with CM-CDs, further confirming the conclusion that the CM-CDs and P-CDs provide complementary results. CM-γ-CD causes enantiomeric differentiation of two of the aryl resonances of **29** that is not observed with the P-CDs. However, the P-β- and P-γ-CDs cause enantiomeric differentiation of aliphatic resonances of **29** that is not observed with the CM-CDs.

**Table 5:** Enantiomeric differentiation in ppm in the ^1^H NMR spectra (400 MHz) of **27**–**29** (10 mM) with P-CDs and CM-CD [31] in D_2_O. The concentration of the cyclodextrin is 20 mM unless otherwise indicated.

		P-α-CD-L_DS_	P-α-CD-H_DS_	P-β-CD-L_DS_	P-β-CD-H_DS_	P-γ-CD-L_DS_	P-γ-CD-H_DS_	CM-CD

**27**	CH_3_	0.004^a^	0.004^a^	0	0	0.004^a^	0.005^a^	
**28**	CH	0	0	0	0.020	0	0	
	N-CH_3_	0	0	0.016^a^	0.011^a^	0.016^a^	0	
	C-CH_3_	0	0	0	0	0.011^a^	0	
	H4	0	0	0	0	0.013^a^	0	
	H8	0	0	0	0	0.011^a^	0	
**29**	N-CH_2_	0	0	0.006^a^	0.008	0.007^b^	0.008^a^	0
	O-CH_2_	0	0	0	0	0	0.006^b^	0
	C-CH_3_	0	0	0.027	0.009	0	0	0
	H2	0	0	0	0	0	0	0.017 - γ
	H8	0	0	0	0	0	0	0.022 - γ

^a^10 mM; ^b^5 mM.

Substrates **30**–**33** are a series of antihistamines that have both an aryl and a pyridyl ring. Of all the compounds examined previously with the CM-CDs, **30**–**33** were noteworthy for both the number of resonances that exhibited enantiomeric differentiation and the magnitude of the distinction. Many resonances exhibit enantiomeric differentiation greater than 0.02 ppm, with one as high as 0.08 ppm, for **30**–**33** with CM-CDs (Table 6). While the P-CDs are not nearly as effective for **30**–**33** as the CM-CDs, there are still eight resonances of **30**–**33** where one of the P-CDs caused larger enantiomeric differentiation than any of the CM-CDs. As with many of the other compounds examined herein, the P-CDs tend to be most effective at causing enantiodifferentiation for the aliphatic resonances.

**Table 6:** Enantiomeric differentiation in ppm in the ^1^H NMR spectra (400 MHz) of **30**–**33** (10 mM) with P-CDs and CM-CD [21,31] in D_2_O. The concentration of the cyclodextrin is 20 mM unless otherwise indicated.

		P-α-CD-L_DS_	P-α-CD-H_DS_	P-β-CD-L_DS_	P-β-CD-H_DS_	P-γ-CD-L_DS_	P-γ-CD-H_DS_	CM-CD

**30**	CH	0	0	0	0	0	0	0.039 - α
	N-CH_2_	0	0	0.010	0.011^a^	0	0	0
	N-CH_2_’	0	0	0.013	0.013^a^	0	0	0
	H4	0	0	0	0	0	0	0.018 - γ
	H3’	0	0	0	0	0	0	0.075 - α
	H4’	0	0	0.004	0.005^a^	0	0.004	0.080 - α
	H6’	0	0	0	0	0	0	0.042 - β
**31**	CH	0	0	0.013	0.008^a^	0.015	0	0.021 - α
	N-CH_3_	0.011	0.016	0	0	0	0	0
	H2	0	0	0.005	0	0	0.005	0.034 - β
	H3	0	0	0.013	0.014	0	0	0.040 - γ
	H3’	0	0	0	0	0.012^a^	0	0.069 - α
	H4’	0	0	0.015	0.015	0	0	0.074 - β
	H5’	0	0	0	0	0	0.017	0
	H6’	0	0	0	0	0	0	0.047 - β
**32**	N-CH_2_	0	0	0.011^a^	0	0	0	0
	O-CH_2_	0.011^a^	0	0	0	0	0	0
	C-CH_3_	0	0.005	0	0.006	0	0	0.021 - β
	H3’	0	0.008	0.009	0.007	0	0	0.010 - γ
	H4’	0	0	0	0	0	0	0.021 - β
	H5’	0	0	0.020	0.021	0	0.004	0
	H6’	0	0	0.018^a^	0.012	0	0	0.013 - β
**33**	CH	0.016^a^	0.019^a^	0	0	0	0.020	0.013 - β
	H2	0.019	0.019	0	0	0	0	0.021 - γ
	H3	0	0	0	0	0	0	0.004 - β
	H4’	0	0	0	0	0	0	0.020 - α
	H6’	0	0	0	0	0	0	0.027 - γ

^a^10 mM.

Earlier studies with the CM-CDs demonstrated the effectiveness of adding paramagnetic lanthanide ions such as praseodymium(III) and ytterbium(III) to enhance the enantiomeric differentiation in the NMR spectra of cationic substrates [29–32]. The lanthanide cation binds to the anionic carboxymethyl group on the CM-CD and the magnetic field of the paramagnetic lanthanide ion perturbs the chemical shifts of the substrate bound in the cyclodextrin cavity by a through-space (pseudocontact) mechanism. The ability of paramagnetic lanthanide ions to improve the enantiomeric differentiation in the spectra of substrates in mixtures with P-CDs was next explored.

In some cases, mixing praseodymium(III) or ytterbium(III) nitrate with the P-CD resulted in the formation of a precipitate. In those cases where the addition of lanthanide(III) nitrates to P-CD–substrate mixtures did not result in the formation of a precipitate, the spectra were often broadened. One reason for the broadening may be caused by the slower exchange within the larger ternary lanthanide–P-CD–substrate complex. Another is because the paramagnetic species shortens the relaxation time of the excited nuclei causing uncertainty broadening.

**Figure 5:**
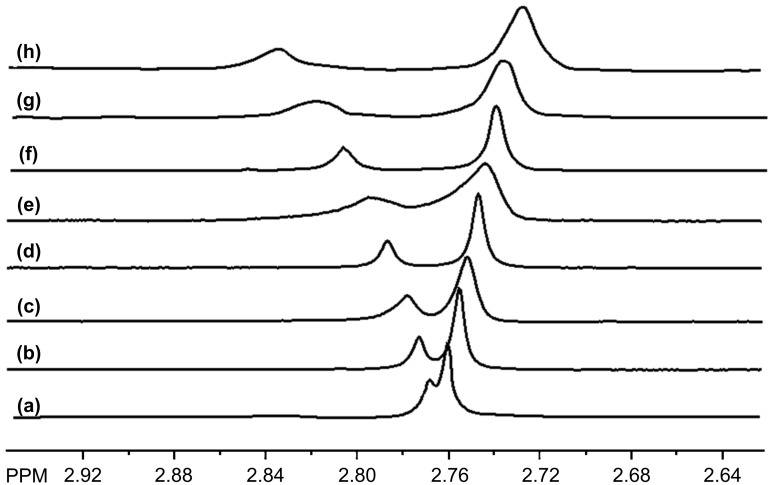
The *N*-methyl resonance of **7** (10 mM, enriched in (1S,2R)-enantiomer) with P-β-CD-L_DS_ (20 mM) and concentrations of ytterbium(III) nitrate of (a) 0 mM, (b) 2 mM, (c) 4 mM, (d) 6 mM, (e) 8 mM, (f) 10 mM, (g) 12 mM and (h) 16 mM.

One example where the addition of a lanthanide ion did produce a large enhancement in enantiomeric differentiation is shown in Figure 5 for the *N*-methyl resonance of **7**. The series of spectra in Figure 5 is for a mixture of **7** (10 mM) and P-β-CD-L_DS_ (20 mM) with increasing concentrations of ytterbium(III) nitrate. The *N*-methyl resonance of **7** with only P-β-CD-L_DS_ shown in Figure 5a exhibits a small degree of enantiomeric differentiation (0.008 ppm). The mixture with ytterbium(III) at 16 mM exhibits an enantiomeric differentiation of 0.109 ppm. An interesting observation is that the resonance of the (1*S*,2*R*)-enantiomer is more shielded on the addition of ytterbium(III) whereas the resonance of the (1*R*,2*S*)-enantiomer is deshielded. The equation that predicts the magnitude of the through-space shifts caused by a lanthanide ion has an angle term that can be either positive or negative depending on the geometry of the complex and the position of a nucleus relative to the principle magnetic axis of the complex. In all likelihood, the behavior seen in the spectra in Figure 5 reflects differences in the sign of this angle term for the two enantiomers [31–32].

## Conclusion

Twenty-three out of the 33 substrates were studied with the P-CDs and CM-CDs. Overall, 54 different resonances exhibited larger enantiomeric differentiation with one of the CM-CDs, whereas 26 different resonances exhibited larger distinction with one of the P-CDs. For 16 of the 23 substrates, there is at least one resonance where larger enantiomeric differentiation was observed with one of the P-CDs. With substrates **3**, **20** and **24**, at least one of the P-CDs caused enantiomeric differentiation whereas none of the CM-CDs was effective. In some cases, it is also possible to add a paramagnetic lanthanide ion such as praseodymium(III) or ytterbium(III) to enhance the enantiomeric differentiation in the ^1^H NMR spectrum of a substrate mixed with a P-CD. Given these observations and the fact that the P-CDs are commercially available, their use as potential chiral NMR reagents for water-soluble cationic compounds is warranted.

## Experimental

### I. Reagents

Sodium salts of phosphated α-, β- and γ-cyclodextrin with low (2–6) and high DS (6–10) were obtained from CarboMer Inc., San Diego, California. The P-CDs were refrigerated between 2–8 °C until use as recommended by the manufacturer. Substrates were obtained from commercial sources either as hydrochloride salts or neutral compounds. Neutral substrates were converted to their hydrochloride salts in deuterium oxide (D_2_O) by adding a slight excess of deuterium chloride (DCl).

### II. Apparatus

Proton (^1^H) NMR spectra were obtained using a Bruker Avance 400 MHz NMR spectrometer. Samples were run in D_2_O with 8 scans at ambient probe temperature.

### III. Procedure

Stock solutions of the P-α-CDs (40 mM), P-γ-CDs (40 mM) and cationic substrates (20 mM), which were enriched in one enantiomer when available, were prepared in D_2_O. P-CD and substrate solutions were kept at ambient temperature. Appropriate aliquots of P-α or P-γ-CD, substrate, and D_2_O were combined in NMR tubes to obtain a 600 μL solution of 20, 10, or 5 mM P-CD and 10 mM substrate. The P-β-CDs were not soluble in D_2_O at 40 mM and 20 mM stock solutions were used in preparing P-β-CD samples at 5 and 10 mM. An appropriate amount of the P-β-CD was weighed for 20 mM solutions.

## Supporting Information

File 1 Complete ^1^H NMR spectra are provided for the samples in Figures 3 and 4. Additional regions of the spectra of Figure 5 are provided.
