# Supplementary material for: Phosphated cyclodextrins as water-soluble chiral NMR solvating agents for cationic compounds
Source: Beilstein J Org Chem. 2017 Jan 6;13:43–53. doi: 10.3762/bjoc.13.6 (PMC5238554; doi:10.3762/bjoc.13.6)
Supplement: File 1 — Complete 1H NMR spectra are provided for the samples in Figures 3 and 4. Additional regions of the spectra of Figure 5 are provided. [file Beilstein_J_Org_Chem-13-43-s001.pdf]

Supporting Information

for

Phosphated cyclodextrins as water-soluble chiral NMR solvating agents for cationic compounds

Cira Mollings Puentes and Thomas J. Wenzel*

Address: Department of Chemistry, Bates College, Lewiston, Maine 04240 USA

Email: Thomas Wenzel - twenzel@bates.edu

*Corresponding author

Complete ^{1}H NMR spectra are provided for the samples in Figures 3 and 4. Additional regions of the spectra of Figure 5 are provided

Figure S1 ^{1}H NMR spectrum of (a) **3** (10 mM, enriched in (*R*)-enantiomer) in the presence of (b) P-α-CD-H (10 mM), (c) P-β-CD-H (10 mM) and (d) P-γ-CD-H (10 mM).

SpinWorks 3: (S)-Dimethyl Phenethyl Amine Substrate

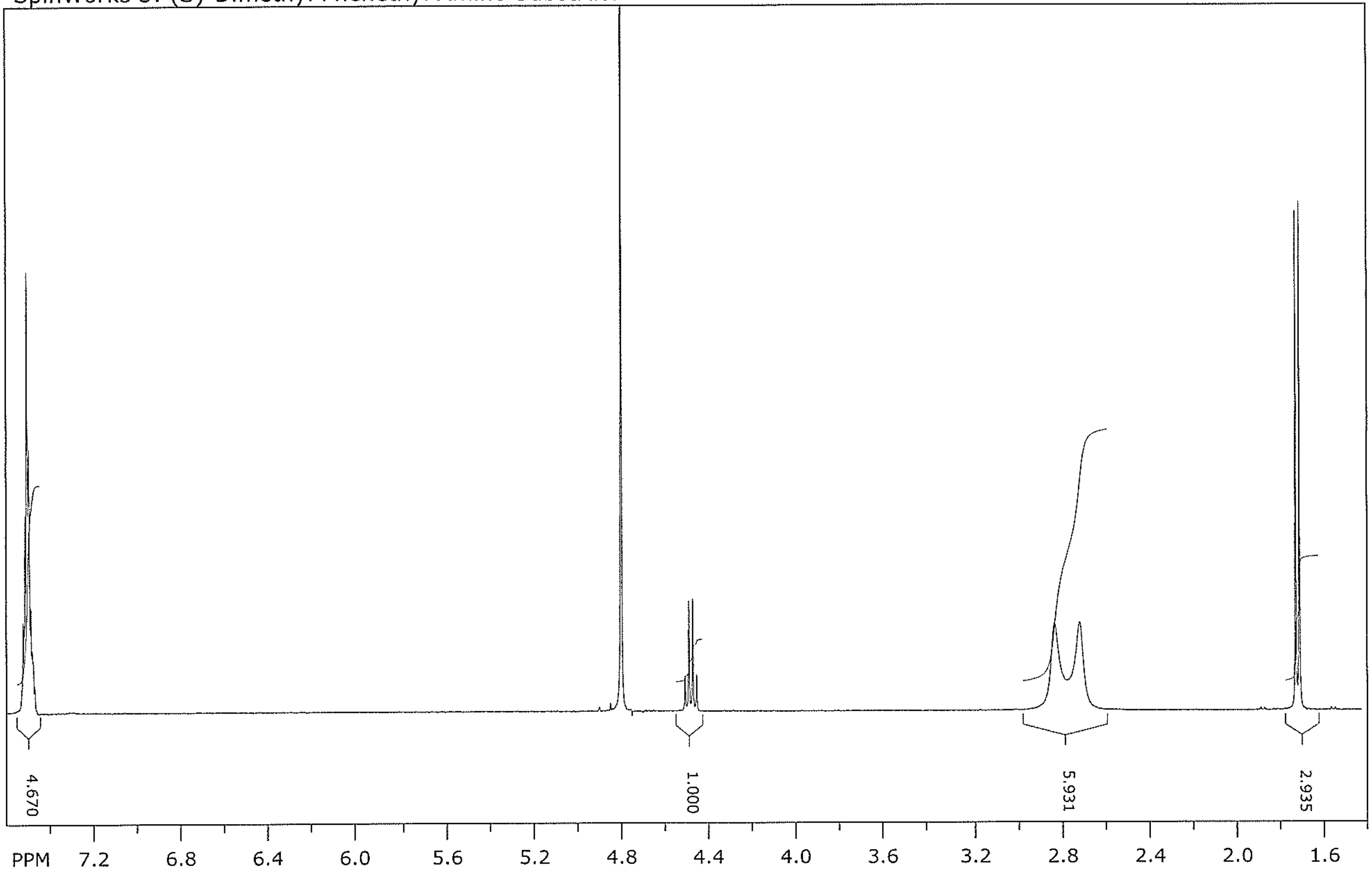

file: I:\bdalvano\nmr\BED_7\1\fid expt: <zg30>
transmitter freq.: 400.132471 MHz
time domain size: 65536 points
width: 8278.15 Hz = 20.6885 ppm = 0.126314 Hz/pt
number of scans: 8

freq. of 0 ppm: 400.129960 MHz
processed size: 32768 complex points
LB: 0.000 GF: 0.0000
Hz/cm: 99.087 ppm/cm: 0.24764

(a)

SpinWorks 3: Phosphated (Alpha-cyclodextrin) HDS (10mM) w/ Dimethylphenethylamine10mM)

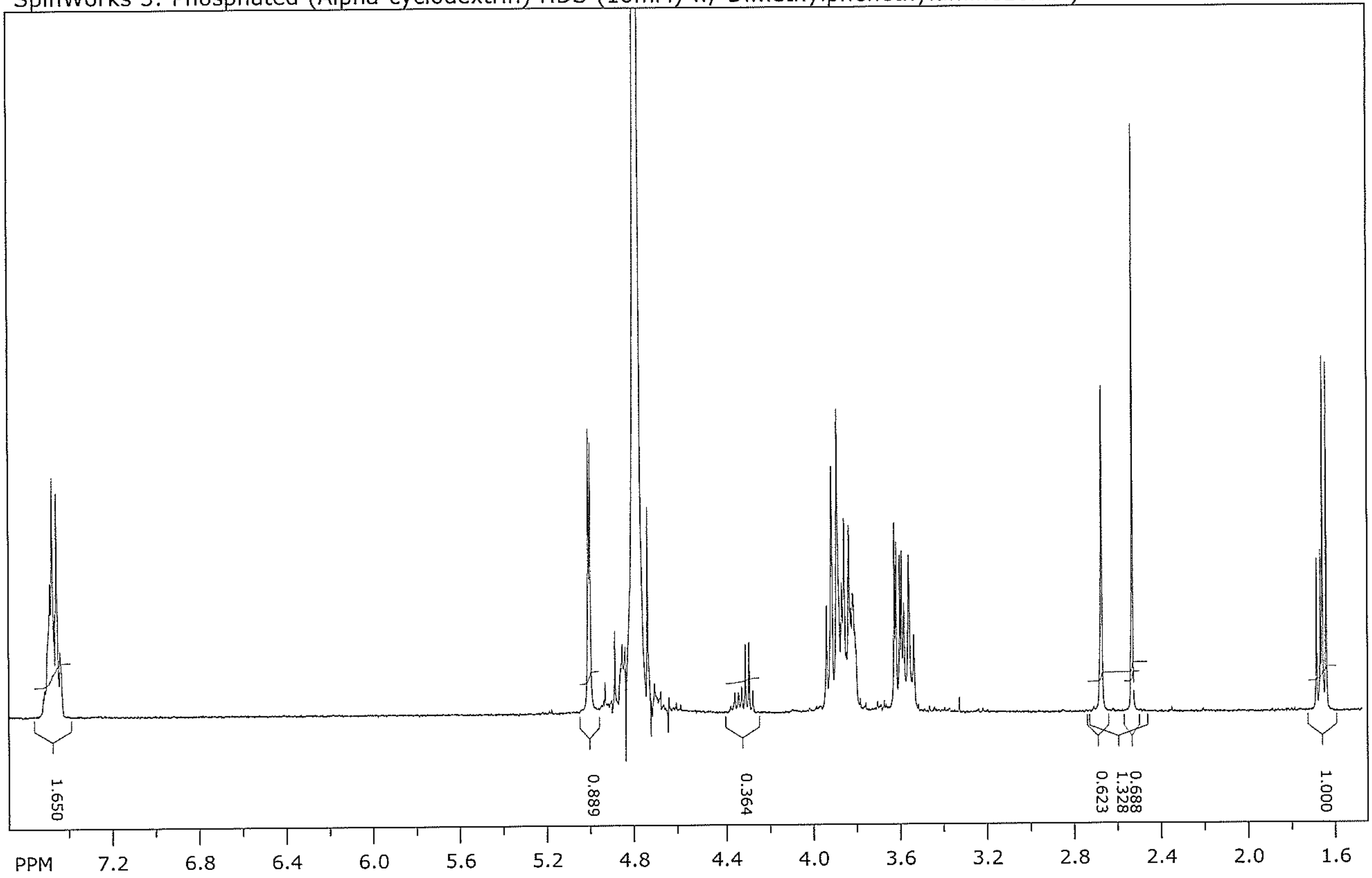

S4 file: ...mollingspuentes\nmr\CMP_390\10\fid expt: <zg30>
transmitter freq.: 400.132471 MHz
time domain size: 65536 points
width: 8278.15 Hz = 20.6885 ppm = 0.126314 Hz/pt
number of scans: 8

freq. of 0 ppm: 400.129960 MHz
processed size: 32768 complex points
LB: 0.000 GF: 0.0000
Hz/cm: 99.589 ppm/cm: 0.24889

(b)

SpinWorks 3: Phosphated (Beta-cyclodextrin) HDS (10mM) w/ Dimethylphenethylamine10mM)

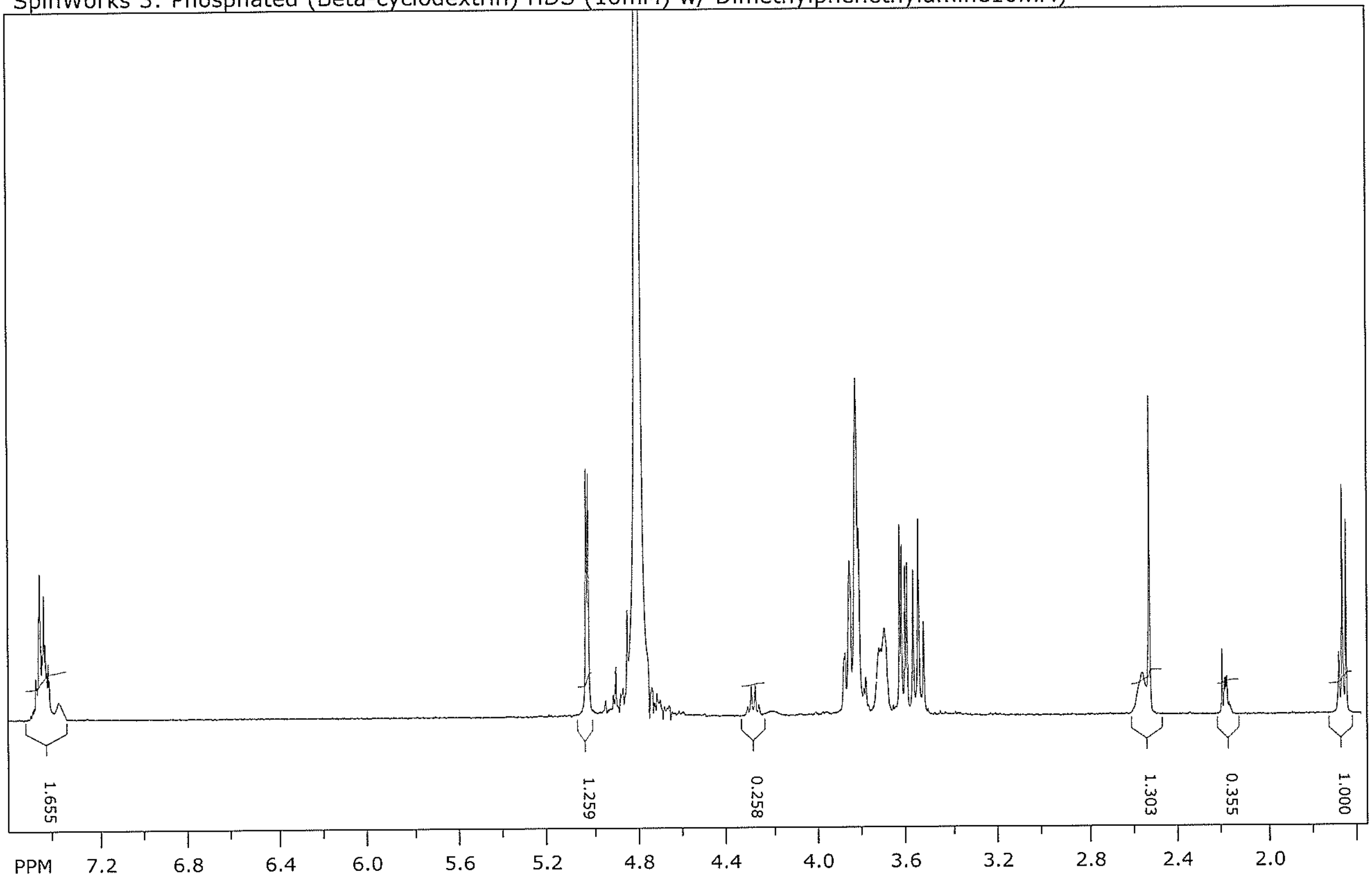

S5 file: ...mollingspuentes\nmr\CMP_392\10\fid expt: <zg30>
transmitter freq.: 400.132471 MHz
time domain size: 65536 points
width: 8278.15 Hz = 20.6885 ppm = 0.126314 Hz/pt
number of scans: 8

freq. of 0 ppm: 400.129960 MHz
processed size: 32768 complex points
LB: 0.000 GF: 0.0000
Hz/cm: 96.765 ppm/cm: 0.24183

(c)

SpinWorks 3: Phosphated (Gamma-cyclodextrin) HDS (10mM) w/ Dimethylphenethylamine10mM)

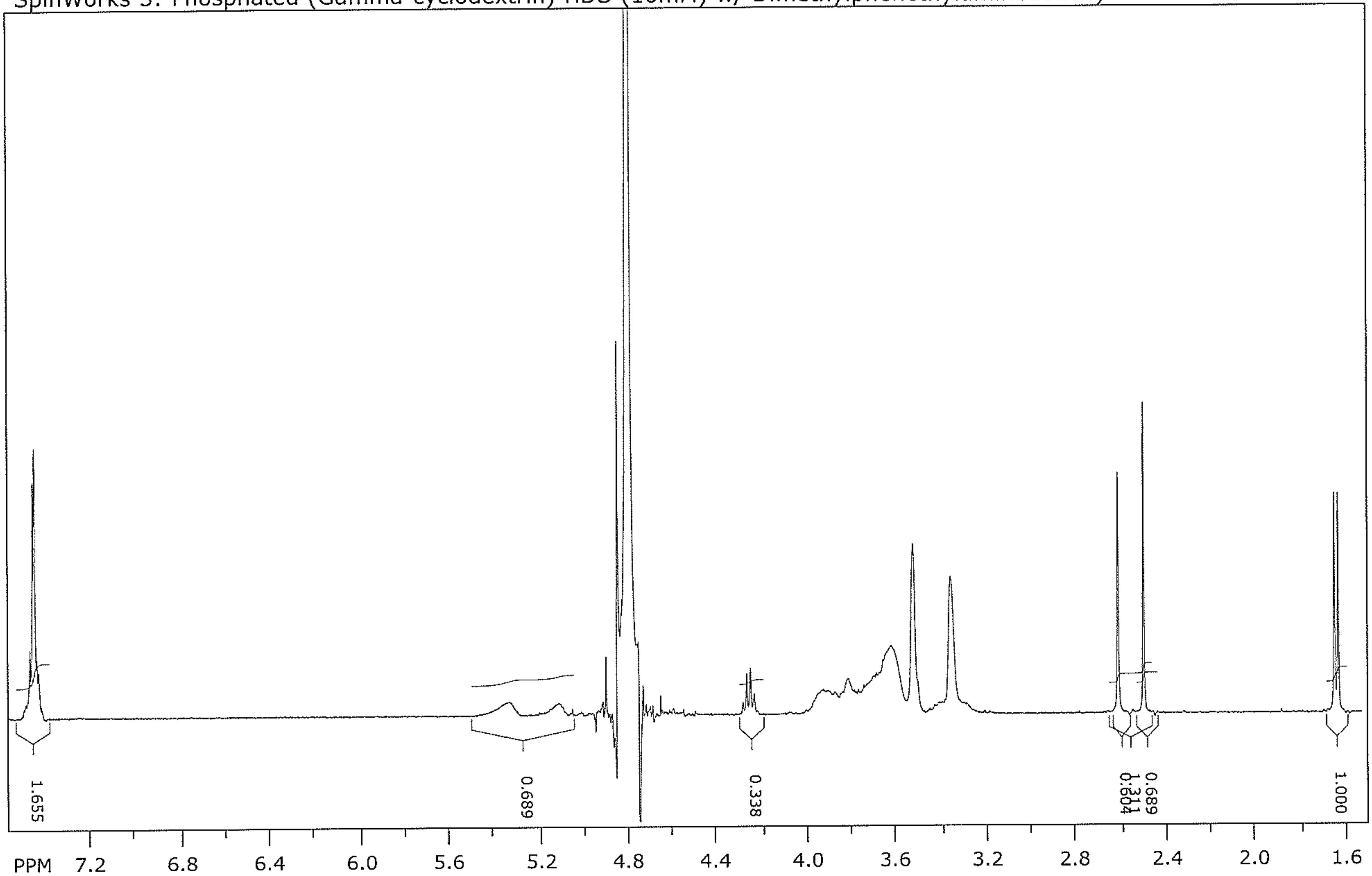

S6

file: ...mollingspuentes\nmr\CMP_394\10\fid expt: <zg30>
transmitter freq.: 400.132471 MHz
time domain size: 65536 points
width: 8278.15 Hz = 20.6885 ppm = 0.126314 Hz/pt
number of scans: 8

freq. of 0 ppm: 400.129960 MHz
processed size: 32768 complex points
LB: 0.000 GF: 0.0000
Hz/cm: 97.099 ppm/cm: 0.24267

(d)

Figure S2. ^{1}H NMR spectrum of (a) **7** (10 mM, enriched in (1*S*2*R*)-enantiomer) with 20 mM of (b) P-α-CD-L, (c) P-α-CD-H, (d) P-β-CD-L, (e) P-β-CD-H, (f) P-γ-CD-L and (g) P-γ-CD-H.

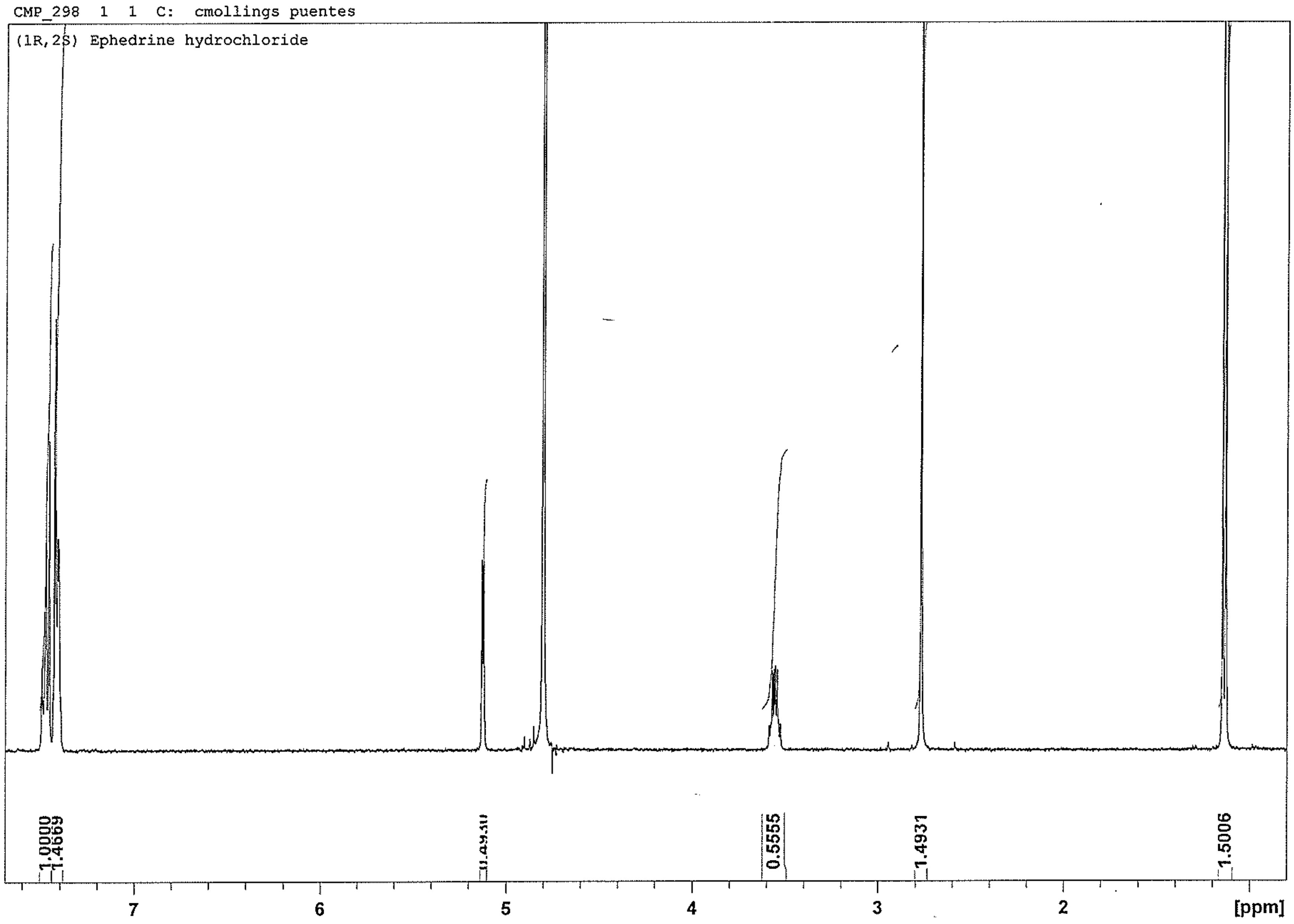
CMP_298 1 1 C: cmollings puentes
(1R,2S) Ephedrine hydrochloride
1.0000
1.4669
0.4950
0.5555
1.4931
1.5006
7
6
5
4
3
2
[ppm]

(a)

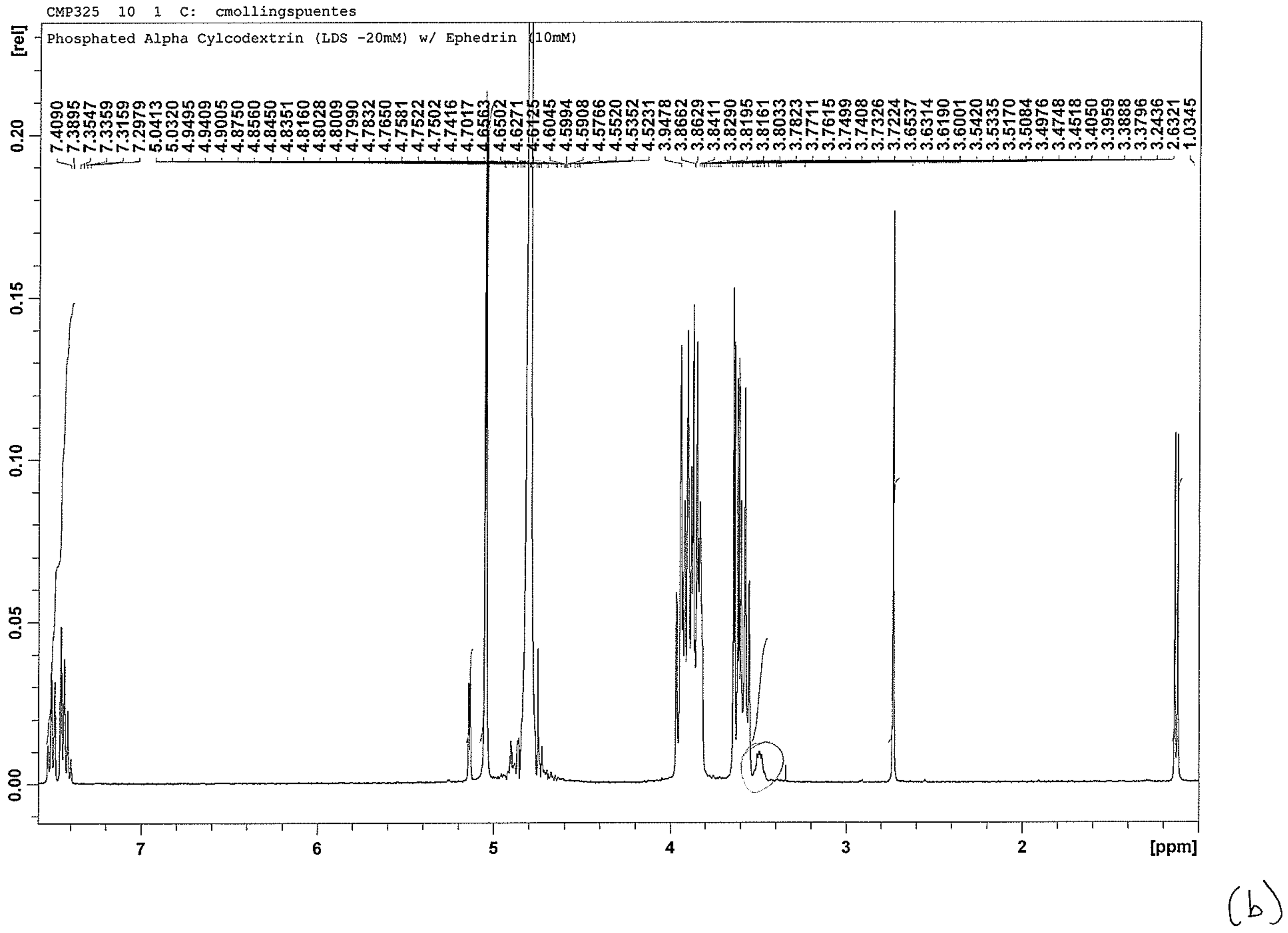
CMP325 10 1 C: cmollingspuentes
Phosphated Alpha Cylcodextrin (LDS -20mM) w/ Ephedrin (10mM)
7.4090
7.3895
7.3547
7.3359
7.3159
7.2979
5.0413
5.0320
4.9495
4.9409
4.9005
4.8750
4.8560
4.8450
4.8351
4.8160
4.8028
4.8009
4.7990
4.7832
4.7650
4.7581
4.7522
4.7502
4.7416
4.7017
4.6563
4.6502
4.6271
4.6125
4.6045
4.5994
4.5908
4.5766
4.5520
4.5352
4.5231
3.9478
3.8662
3.8629
3.8411
3.8290
3.8195
3.8161
3.8033
3.7823
3.7711
3.7615
3.7499
3.7408
3.7326
3.7224
3.6537
3.6314
3.6190
3.6001
3.5420
3.5335
3.5170
3.5084
3.4976
3.4748
3.4518
3.4050
3.3959
3.3888
3.3796
3.2436
2.6321
1.0345
[rel]
0.20
0.15
0.10
0.05
0.00
7
6
5
4
3
2
[ppm]
(b)

(c)

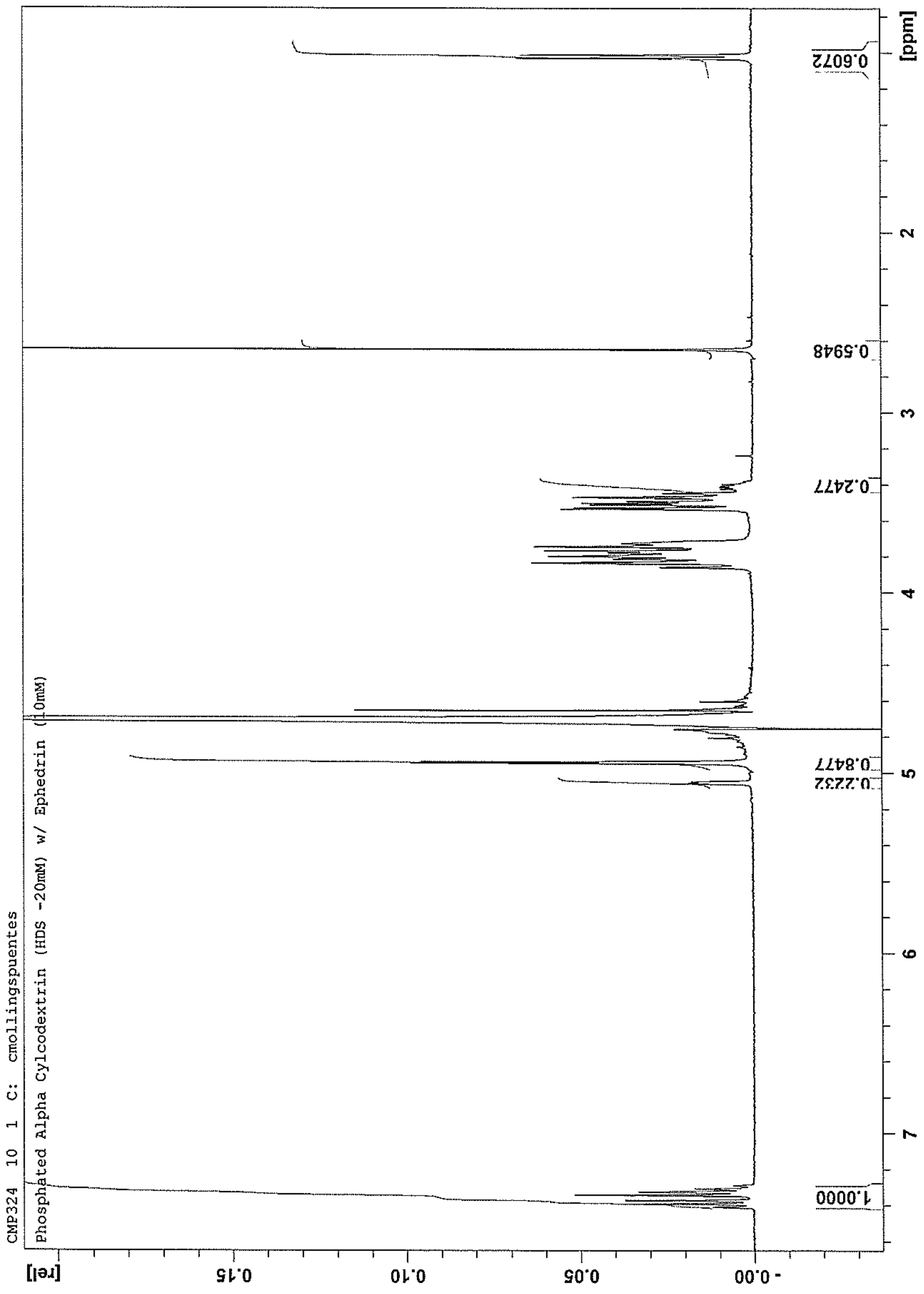

CMP324 10 1 C: cmollingspuentes
Phosphated Alpha Cylcodextrin (HDS -20mM) w/ Ephedrin (10mM)
1.0000
0.2232
0.8477
0.2477
0.5948
0.6072
[ppm]
7
6
5
4
3
2
[rel]
0.15
0.10
0.05
0.00

(2)

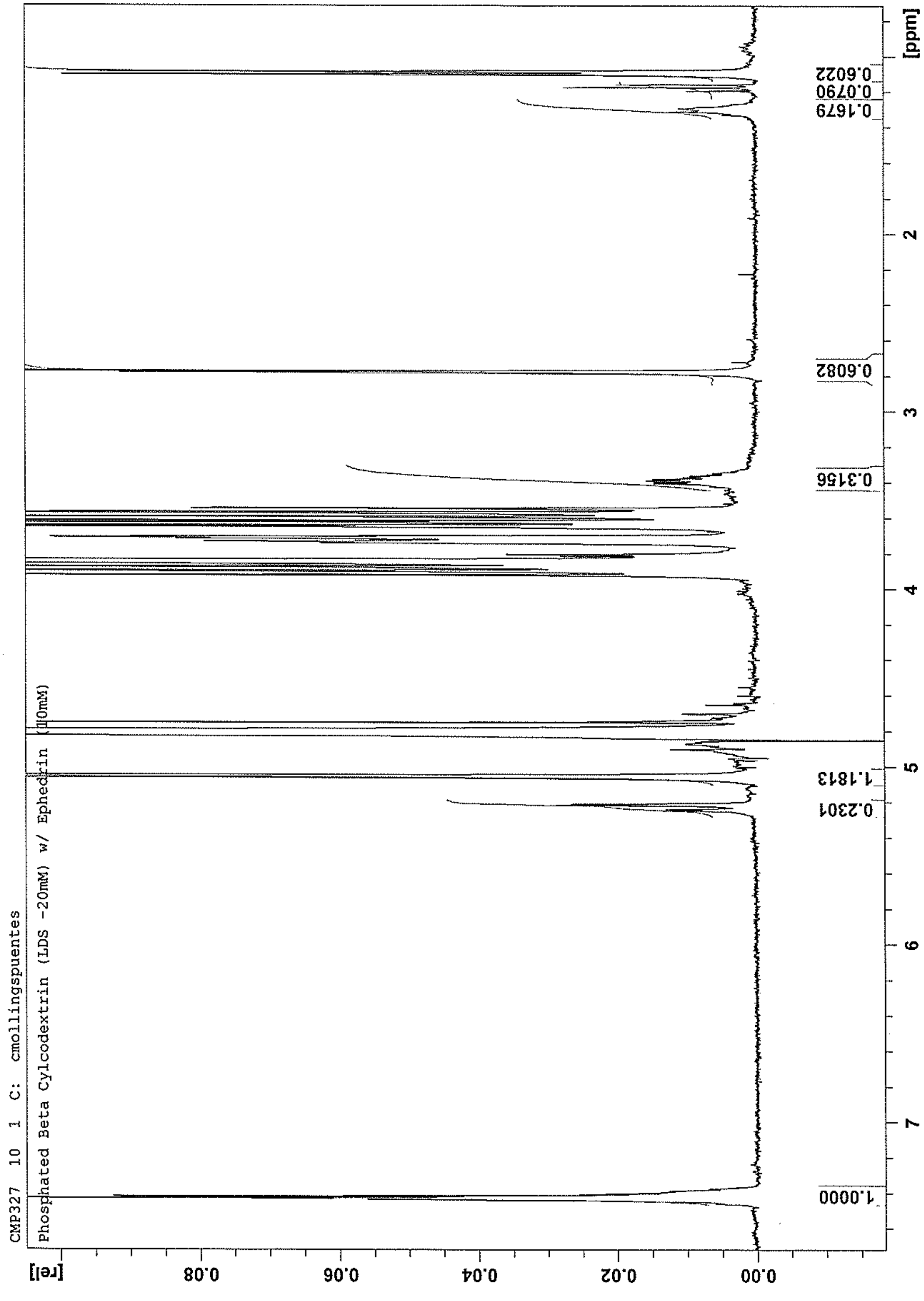
CMP327 10 1 C: cmollingspuentes
Phosphated Beta Cylcodextrin (LDS -20mM) w/ Ephedrin (10mM)
1.0000
0.2301
1.1813
0.3156
0.6082
0.1679
0.0790
0.6022
7
6
5
4
3
2
[ppm]
0.00
0.02
0.04
0.06
0.08
[rel]

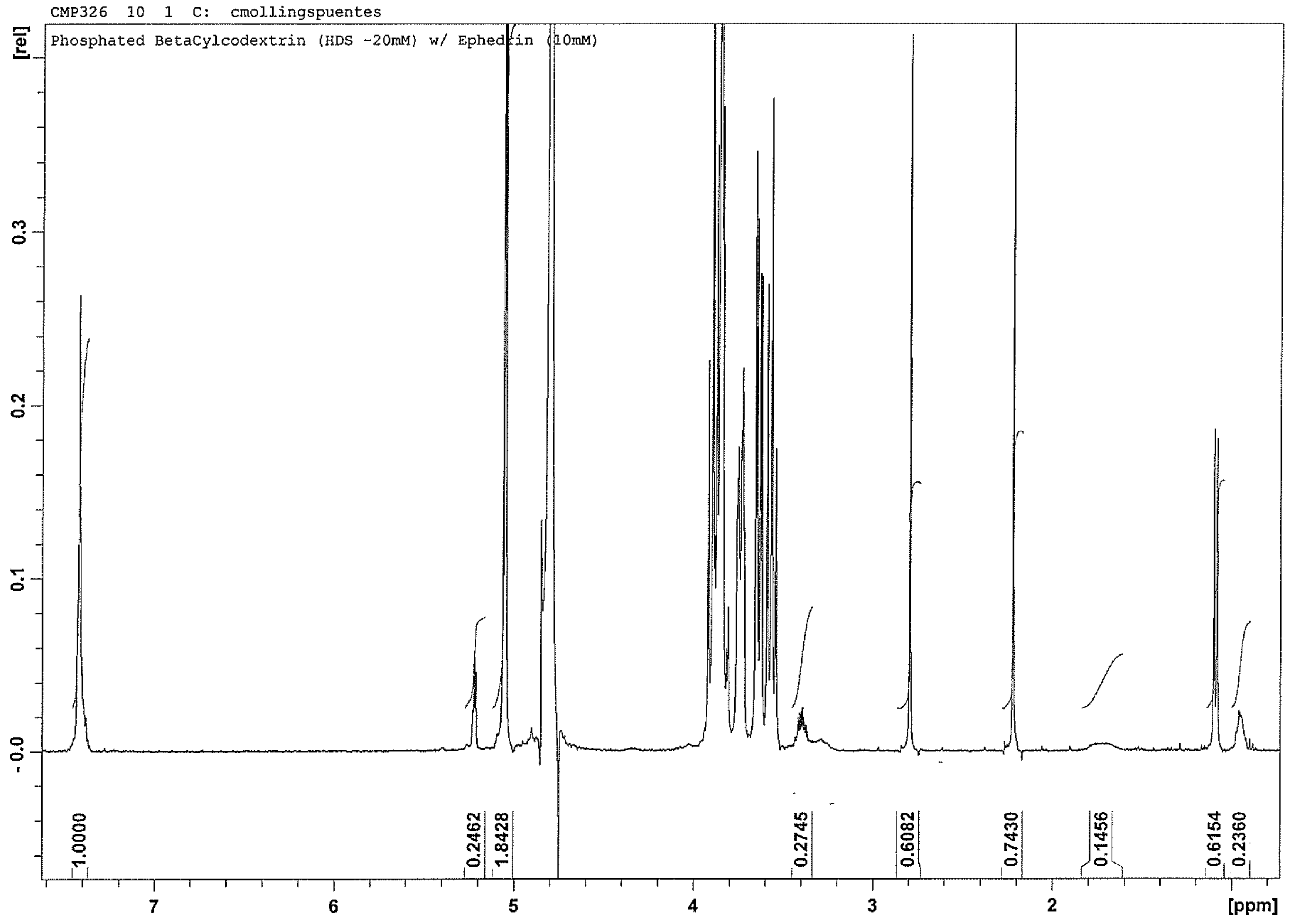
CMP326 10 1 C: cmollingspuentes
Phosphated BetaCylcodextrin (HDS ~20mM) w/ Ephedrin (10mM)
[rel]
0.3
0.2
0.1
-0.0
1.0000
0.2462
1.8428
0.2745
0.6082
0.7430
0.1456
0.6154
0.2360
7
6
5
4
3
2
[ppm]

(e)

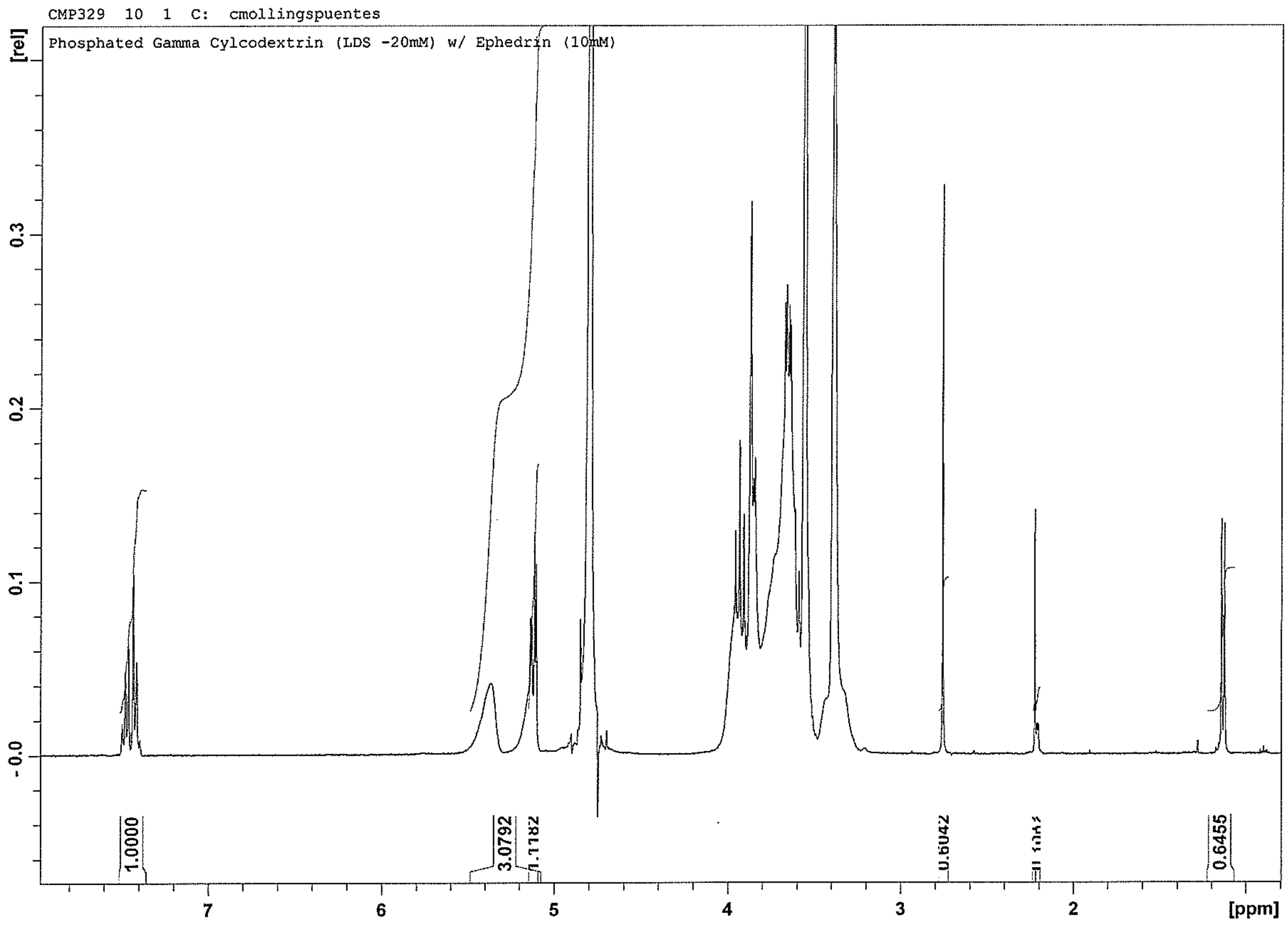
CMP329 10 1 C: cmollingspuentes
Phosphated Gamma Cylcodextrin (LDS -20mM) w/ Ephedrin (10mM)
[rel]
0.3
0.2
0.1
-0.0
1.0000
3.0792
1.1182
0.6042
[illegible]
0.6455
7
6
5
4
3
2
[ppm]

(F)

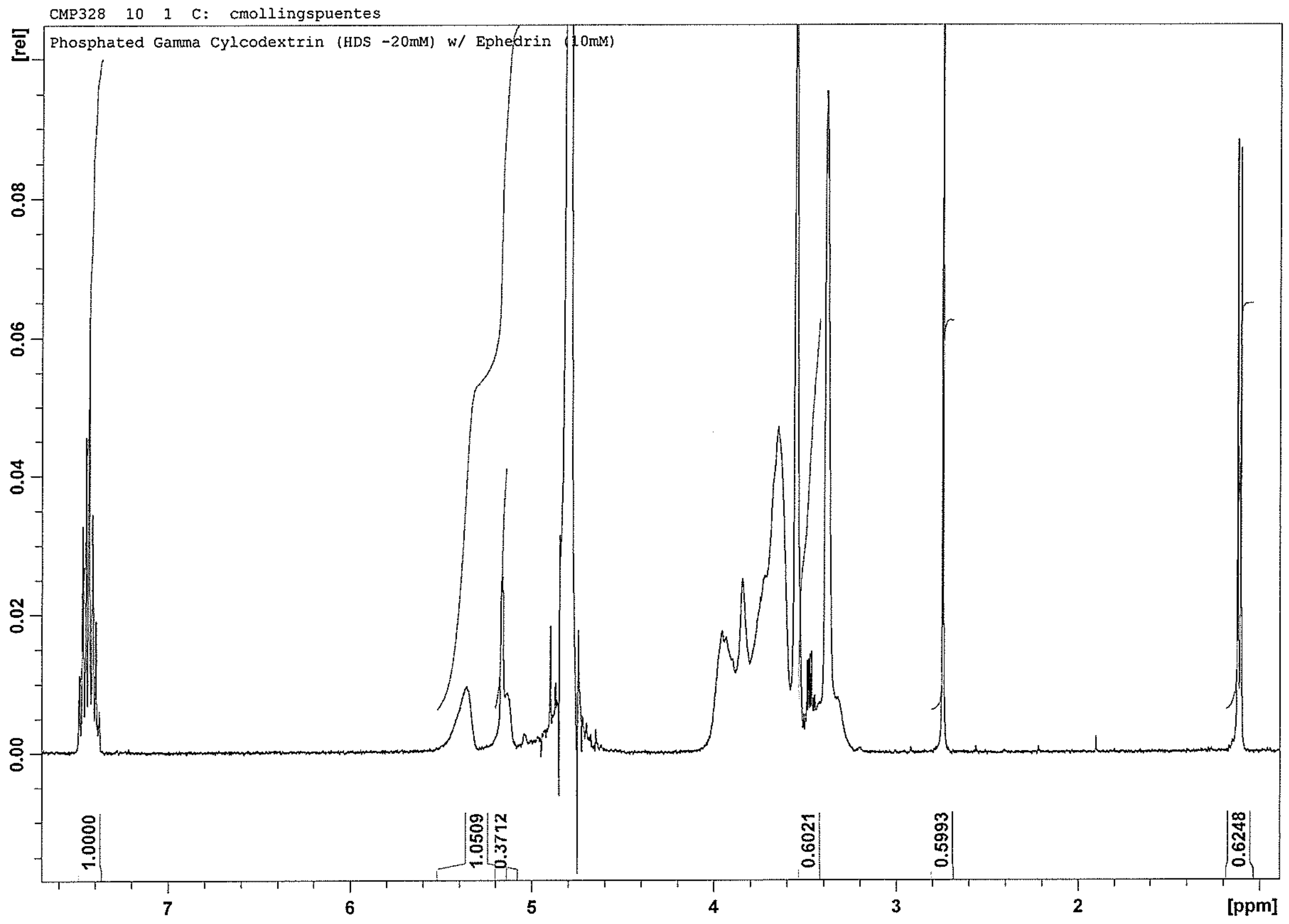
CMP328 10 1 C: cmollingspuentes
Phosphated Gamma Cylcodextrin (HDS -20mM) w/ Ephedrin (10mM)
[rel]
0.08
0.06
0.04
0.02
0.00
1.0000
1.0509
0.3712
0.6021
0.5993
0.6248
7
6
5
4
3
2
[ppm]

(9)

Figure S3. Regions of the ^{1}H NMR spectrum of **7** (10 mM, enriched in (1*S*2*R*)-enantiomer) with P-β-CD-L (20 mM) and increasing concentrations of ytterbium(III)nitrate.

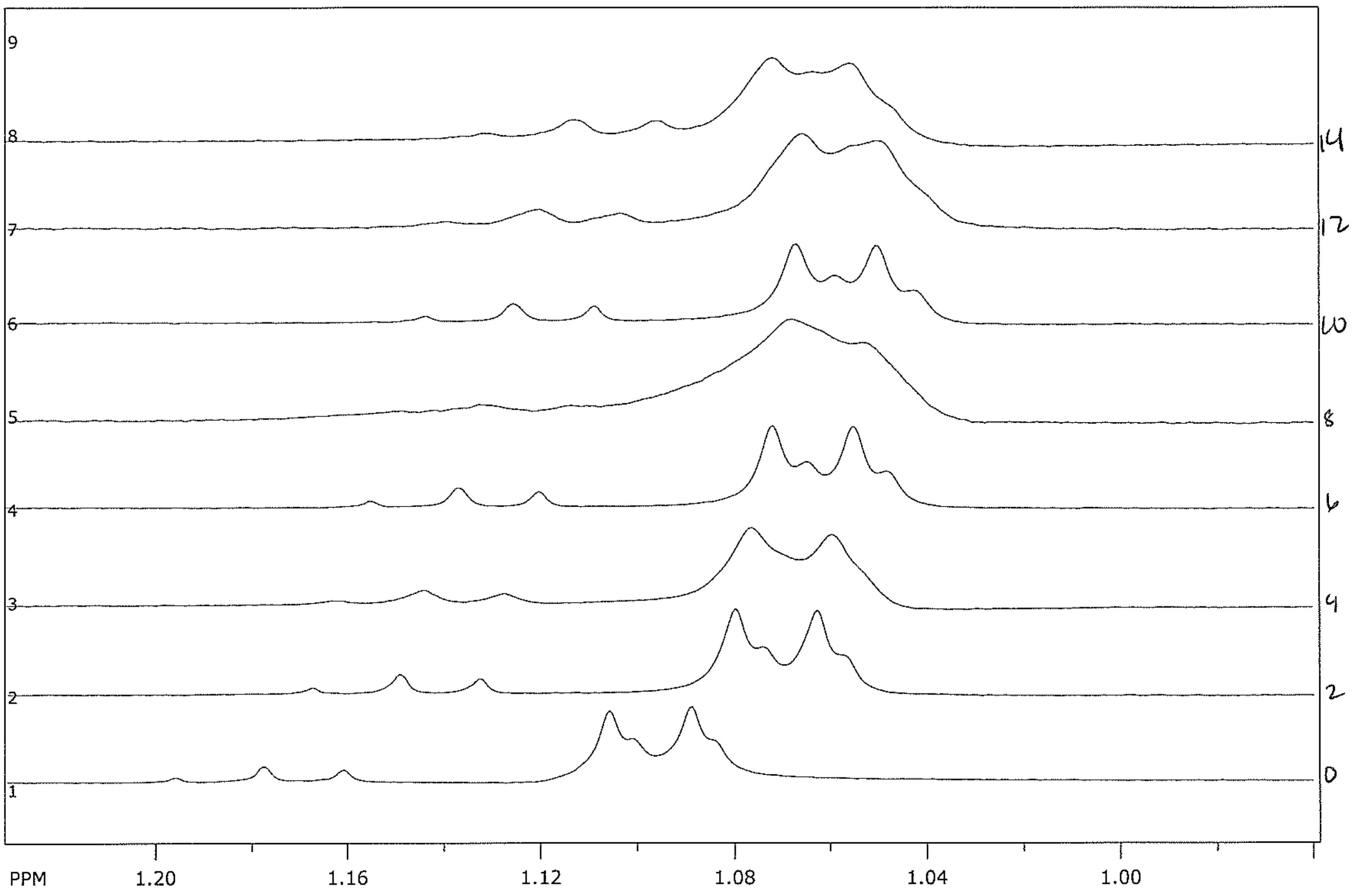

Figure 6 Ephedrine 10mM with 20mM BCD LDS and Yb(NO3)3 (2-20 mM)

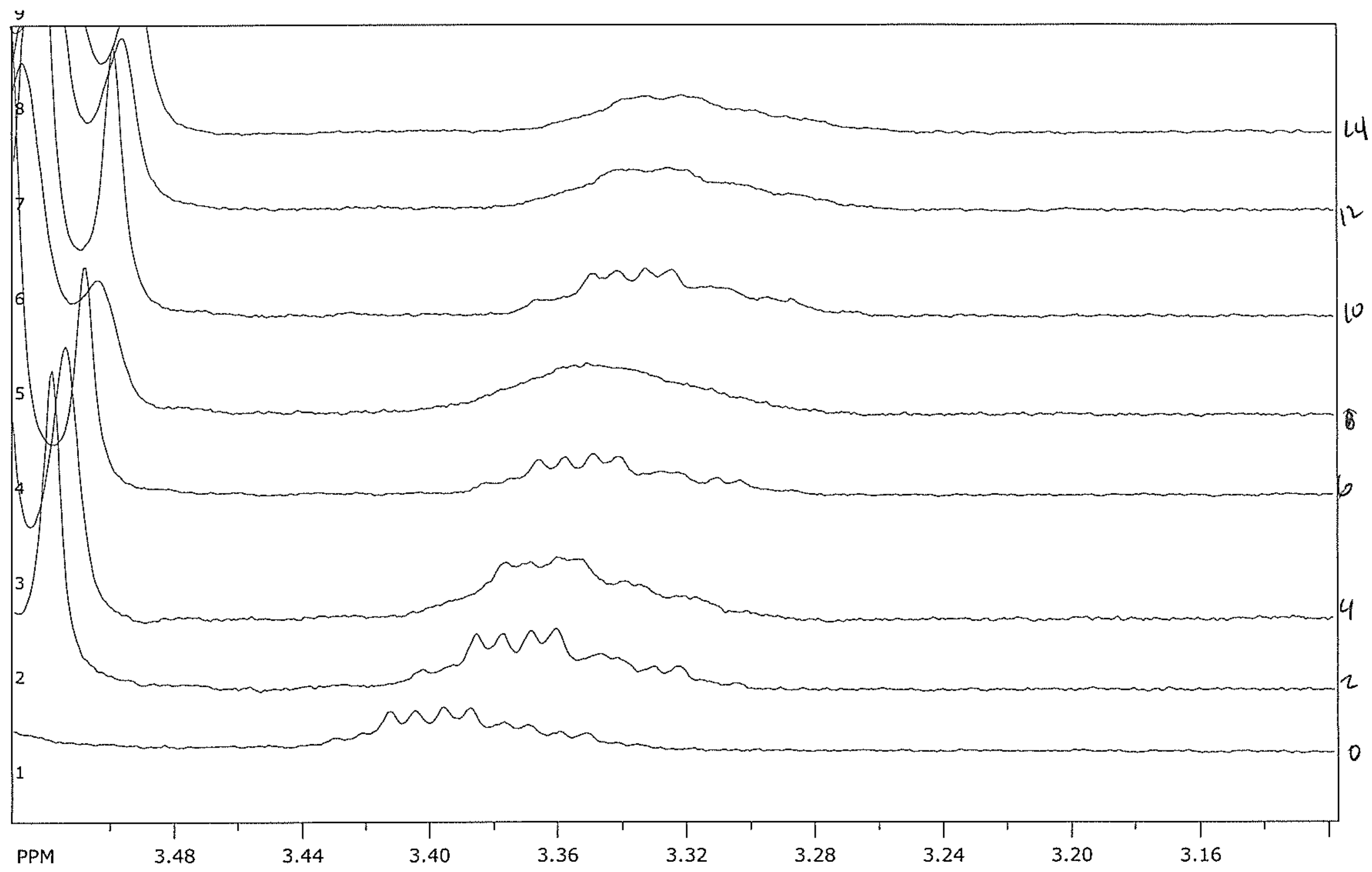

Figure 8 Ephedrine 10mM with 20mM BCD LDS and Yb(NO3)3 (2-20 mM)

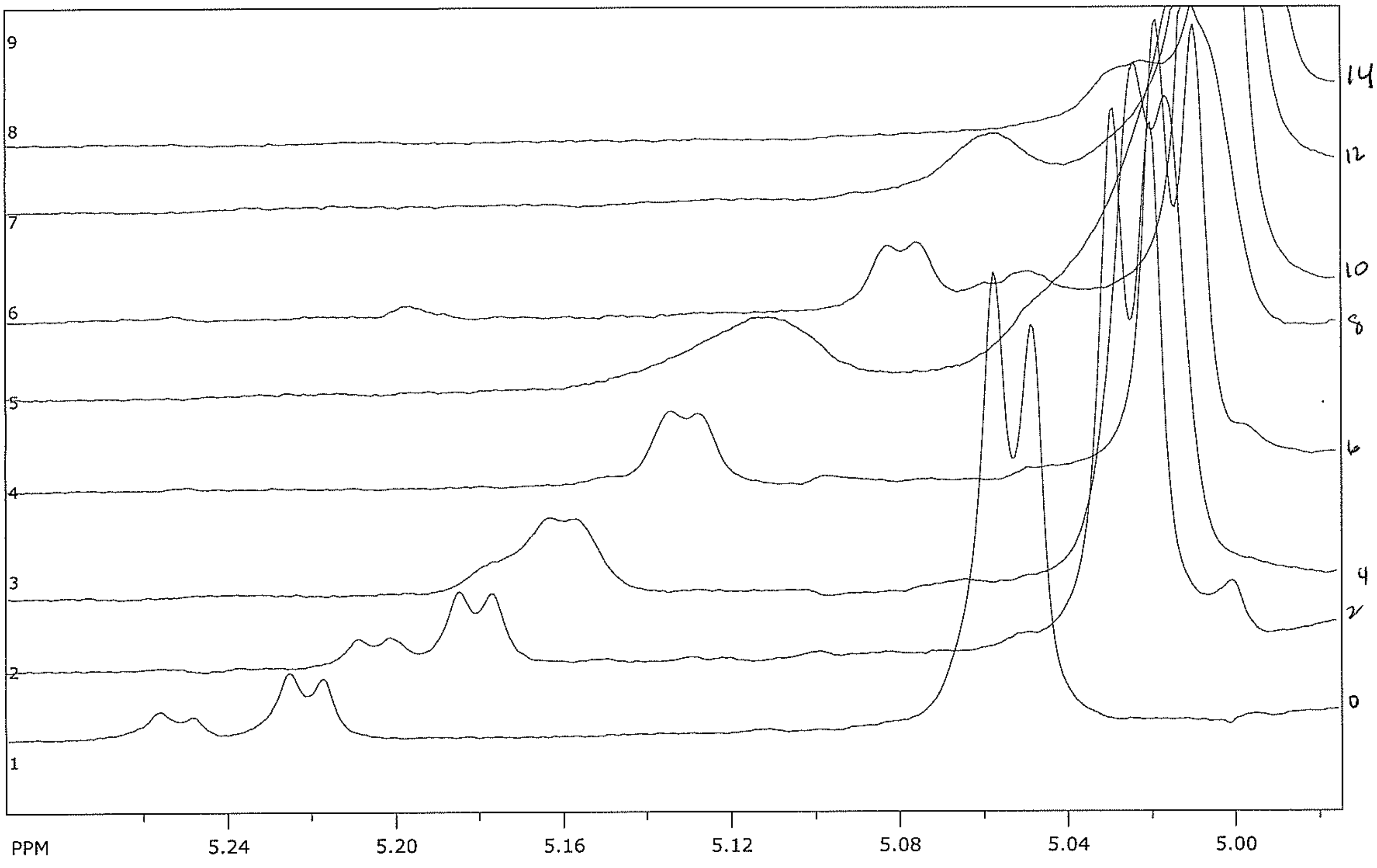

Figure 7 Ephedrine 10mM with 20mM BCD LDS and Yb(NO3)3 (2-20 mM)
